# Air bubble dispersion imaging in whipping cream with specified fat contents by fuzzy phase classification implemented in electrical impedance tomography

**DOI:** 10.1038/s41598-025-03773-3

**Published:** 2025-05-29

**Authors:** Songshi Li, Prima Asmara Sejati, Ryuichi Fukumoto, Masahiro Takei

**Affiliations:** 1 Graduate School of Engineering, Chiba University, 1-33, Yayoicho, Inage-ku, Chiba-shi, 263-8522 Chiba Japan; 2 Department of Electrical Engineering and Informatics, Sekip Unit III, Bulaksumur, Vocational College, Universitas Gadjah Mada, Yogyakarta, 55281 Indonesia; 3 Graduate School of Science and Engineering, Chiba University, 1-33, Yayoicho, Inage-ku, Chiba-shi, 263-8522 Chiba Japan

**Keywords:** Whipping cream, Electrical impedance tomography, Fuzzy phase classification, Air bubble dispersion, Fat content, Internal morphological structure, Imaging techniques, Characterization and analytical techniques

## Abstract

The air bubble dispersion in whipping cream during agitation has been imaged by fuzzy phase classification implemented in electrical impedance tomography (*f*EIT) for internal morphological structure visualization. The *f*EIT consists of non-linear conductivity $\:\sigma\:$ reconstruction and fuzzy phase classification to classify $\:\sigma\:$ into a probabilistic cluster $\:{u}_{j}$ ($\:j=1$: oil-in-water phase, $\:j=2$: air bubble). In the experiments, probabilistic air bubble cluster $\:{u}_{2}$ of whipping cream with five milk fat (MF) contents were reconstructed by *f*EIT during agitation to image the air bubble dispersion. The $\:{u}_{2}$ demonstrated the air bubble dispersion within the sensor area caused by agitation. The spatial average $\:\langle{u}_{2}\rangle$ was increased and then decreased due to the incorporation and subsequent departure of air bubbles during agitation. The peak $\:{\langle{u}_{2}\rangle}_{\:}$ time *t*_*s*_, which represents the critical saturation point of air bubbles, was decreased as MF content increased, which was verified by OR measurement and microscope observation. Compared to $\:\sigma\:$ reconstructed by EIT influenced by the liquid water phase, fat and air bubbles, $\:\langle{u}_{2}\rangle$ reconstructed by *f*EIT successfully imaged air bubble dispersion in whipping cream. Furthermore, *f*EIT demonstrated comparable performance to OR measurements in evaluating the internal morphological structure of whipping cream during agitation, while providing the advantage of inline measurement.

## Introduction

Whipping cream is a complex oil-in-water (O/W) emulsion composed primarily of a liquid water phase, fat globules and air bubbles^1^. During the agitation process, air bubbles are introduced into the liquid cream, which causes the whipping cream to transition into a semi-solid state^2^. The agitation process involves four distinct stages of internal morphological evolution, which are (1) the introduction of large and fragile air bubbles, (2) the aggregation of fat globules which adhere to the air bubble surface, (3) the stable dispersion of air bubbles supported by a three-dimensional fat chain network and (4) the homogenization and shrinkage of microstructure, which ultimately results in a solid-like structure^3^. The internal morphological structure, which affects the physical properties of whipping cream^4^, is strongly influenced by fat content, as fat plays a pivotal role in stabilizing air bubble size and dispersion which is referred to as air bubble stability. As the fat content is increased, enhanced air bubbles stability facilitates a more uniform and earlier formation of the air bubble phase, which is highly considered to be monitored through air bubble dispersion characterized by partially coalesced fat layers surrounding the air bubbles during agitation^5^. Higher fat content enhances air bubble stability, which forms stronger partially coalesced fat layers that accelerate the transition to a solid-like structure. This transition influences the time required to reach the critical saturation point of air bubbles and ultimately affects the quality of final products such as cakes, pastries, ice cream, and desserts^6^. Consequently, imaging air bubble dispersion during the agitation process is crucial for understanding the internal morphological structure of whipping cream.

Commonly, the overrun (*OR*) to quantify air holdup^7^, rheological parameters to describe the elastic modulus $\:{G}^{{\prime\:}}\:$and viscous modulus $\:{G}^{{\prime\:\prime\:}}$^8^ and optical parameters to capture the air bubble size with a scanning electron microscope (SEM)^5^ are frequently applied to evaluate the air bubble dispersion of whipping cream for understanding the internal morphological structure^9^. The maximum value of *OR* indicates the critical saturation point of air holdup, which represents the maximum air bubble stability achieved during the agitation process^10^. Besides, the rheological parameters, measured by a controlled-stress rheometer, provide insights into the viscoelastic properties arising from air bubble dispersion (Ghorbani‐HasanSaraei et al. 2019). Then, the optical parameters visualize air bubble dispersion, which offered detailed information on internal morphological structure and cream formation processes^11^. Although these studies have demonstrated the effectiveness of evaluating air bubble dispersion in whipping cream, applying these methods as on-line measurements during the agitation process faces challenges in achieving higher levels of quality control.

In order to provide on-line air bubble dispersion of whipping cream evaluation, an electrical impedance spectroscopy (EIS) has been applied, which gain attention for the rapid response and non-invasiveness in measuring impedance spectral over frequency and analyzing electrical characteristics^12^. Currently, EIS is applied to many different industrial fields, such as agitation process of lithium-ion battery slurry^13^, inhomogeneous bubbly flow fields^14^, particle-liquid centrifugal fields^15^ and high-temperature molten salt fields^16^. Compared to *OR*, rheological properties, and microscopic images, EIS offers greater adaptability for on-line evaluation of whipping cream, as the dielectric relaxation effectively reflects the changes in mechanical properties^17^. Nevertheless, the spectral data obtained from EIS struggles to capture spatial information, particularly the inhomogeneity of the internal morphological structure. In order to address the spatial limitation of EIS, electrical impedance tomography (EIT) has been applied to understand the internal morphological structure by imaging the spatial distribution of the electrical properties^18^. Nevertheless, conventional EIT struggles to accurately interpret the internal morphological structure from conductivity distributions, especially the air bubble dispersion in liquid water phase.

In order to address the challenge of imaging air bubble dispersion in whipping cream by EIT, an unsupervised machine learning method which incorporates fuzziness is proposed for estimating changes in internal morphological structure based on the microstructure distribution and inhomogeneity^19^. The primary structural phases of whipping cream are categorized into two phases: the oil-in-water (O/W) phase which consists of water and dispersed fat globules, and the air bubble phase. Since the conductivity of the O/W phase is approximately $\:{10}^{-1}$ S/m and that of the air bubble phase is around $\:{10}^{-15}$ S/m, which allows a clear differentiation of the two phases by focusing on conductivity distribution and variance. In this study, fuzzy phase classification implemented in electrical impedance tomography (*f*EIT) is proposed to image air bubble dispersion with various fat contents during the agitation process.

The research objectives of this study are as follows:

1. To propose *f*EIT by implementing fuzzy phase classification in electrical impedance tomography.
2. To image air bubble dispersion during agitation by the proposed *f*EIT.
3. To investigate the effect of milk fat content on the air bubble dispersion in whipping cream by comparing the *f*EIT results with *OR* and microscopy images.

## Fuzzy phase classification implemented in electrical impedance tomography (fEIT)

**Fig. 1:**
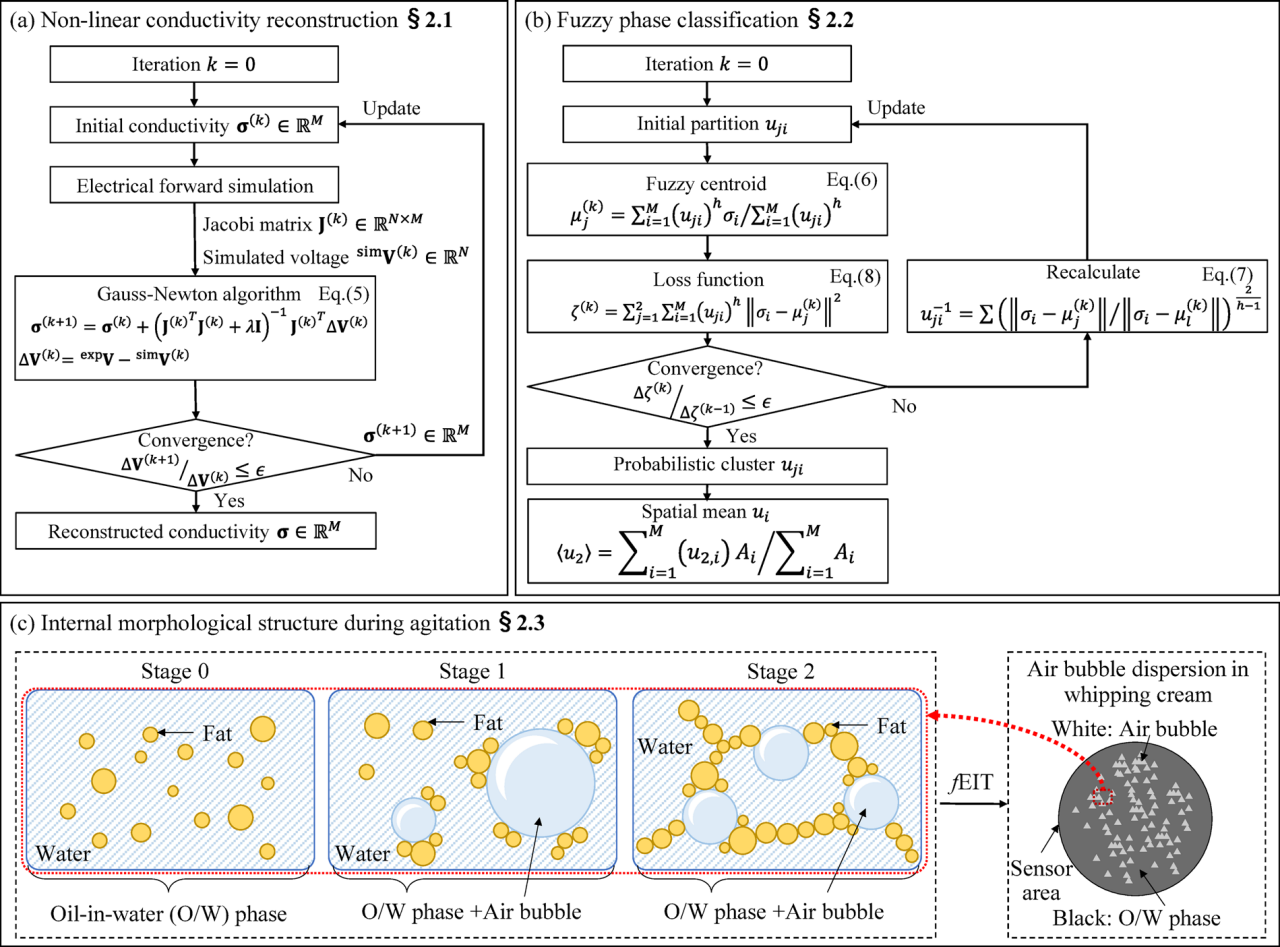
Fuzzy phase classification implemented in electrical impedance tomography (*f*EIT). (**a**) Non-liner conductivity reconstruction. (**b**) Fuzzy phase classification. (**c**) Internal morphological structure during agitation.

Figure 1 shows the conceptual figure of fuzzy phase classification implemented in electrical impedance tomography (*f*EIT) where fuzzy phase classification is employed as an unsupervised machine learning method to estimate air bubble dispersion during agitation, which overcomes the challenges of accurately imaging air bubble dispersion and the ambiguity of the boundary interface between oil-in-water (O/W) and air bubble phases due to the limited electrical measurements. The *f*EIT consists of (a) non-linear conductivity reconstruction and (b) fuzzy phase classification. In the non-linear conductivity reconstruction, the conductivity distribution $\:\sigma\:$ is reconstructed from the experimental voltage $\:{}_{\:}{}^{\text{e}\text{x}\text{p}}V$ and Jacobi matrix $\:\mathbf{J}$ iteratively. The $\:\mathbf{J}$ is computed from the electrical forward simulation and updated after each iteration to ensure a more optimal inverse problem-solving result. In the fuzzy phase classification, the phase classification of the sensor area is probabilistically determined with centroid of cluster $\:{u}_{j}$ based on $\:\sigma\:$. With (a) non-linear conductivity reconstruction and (b) fuzzy phase classification, the air bubble dispersion of whipping cream during agitation is imaged.

### Non-linear conductivity reconstruction

Figure 1 (a) shows the process of non-linear conductivity reconstruction which consists of the electrical forward simulation to calculate $\:\mathbf{J}$ and the inverse problem-solving to reconstruct $\:\sigma\:$. Firstly, mesh segmentation is defined according to the actual geometry of the mixing container and the arrangement of electrodes. The $\:\mathbf{J}\in\:{\mathbb{R}}^{N\times\:M}$ ($\:N$: number of measurement patterns, $\:M$: number of mesh) is computed by a forward problem simulation and to build the conductivity reconstruction model^20^. The governing equation for the quasi-stationary electric field is described as the following Laplace equation with the assumed $\:\sigma\:$ for a known spatial field and the potential distribution $\:\varphi\:$ of in spatial domain $\:{\Omega\:}$.

1 $$\:\nabla\:\bullet\:\left(\sigma\:\nabla\:\varphi\:\right)=0\:\text{i}\text{n}\:{\Omega\:}\:$$

The Neumann boundary condition is applied to each of $\:{{\Gamma\:}}_{wall}$ and $\:\:{{\Gamma\:}}_{electrode}$, with the wall and electrode surfaces as constraint conditions.

2 $$\:\left\{\begin{array}{c}\sigma\:\nabla\:\varphi\:\bullet\:n=\frac{I}{S}\:\:on\:{{\Gamma\:}}_{electrode}\\\:\nabla\:\varphi\:\bullet\:n=0\:\:on\:{{\Gamma\:}}_{wall}\end{array}\right.$$

where $\:I$ is the injected current, *S* is the surface area, and **n** is the surface vector. Based on Eqs. (1) and (2), each element of $\:\mathbf{J}$ is calculated as the sensitivity of the potential difference due to changes in $\:\sigma\:$ with the following equation^21^,

3 $$\:\frac{\partial\:{V}_{\:}}{\partial\:{\sigma\:}_{i}}=-\frac{1}{I}{\int\:}_{{{\Omega\:}}_{i}}^{\:}\nabla\:{V}_{{\Phi\:}}\nabla\:{V}_{{\Psi\:}}d{\Omega\:}$$

where $\:{{\Omega\:}}_{i}$ is the area of $\:i$-th mesh, and $\:{V}_{{\Phi\:}}$ and $\:{V}_{{\Psi\:}}$ are the potentials in the nodes in $\:i$-th mesh with corresponding current-applying electrode pairs. Then, the inverse problem is solved to reconstructed $\:\varvec{\upsigma\:}\in\:{\mathbb{R}}^{M}$ from $\:\mathbf{J}$ and the experimental voltage $\:{}_{\:}{}^{\text{e}\text{x}\text{p}}\mathbf{V}\in\:{\mathbb{R}}^{N}$ as follows:

4 $$\:{}_{\:}{}^{\text{e}\text{x}\text{p}}\mathbf{V}=\mathbf{J}\sigma\:$$

The Gauss-Newton method is utilized to solve the inverse problem iteratively with the algorithm as follows:

5 $$\:{\varvec{\upsigma\:}}^{\left(k+1\right)}={\varvec{\upsigma\:}}^{\left(k\right)}+{\left({{\mathbf{J}}^{\left(k\right)}}^{T}{\mathbf{J}}^{\left(k\right)}+\lambda\:\mathbf{I}\right)}^{-1}{{\mathbf{J}}^{\left(k\right)}}^{T}\varDelta\:{\mathbf{V}}^{\left(k\right)}$$

where, $\:k$ is the number of iterations, $\:\mathbf{I}\in\:{\mathbb{R}}^{M\times\:M}$ is the identity matrix, $\:{\varDelta\:\mathbf{V}}^{\left(k\right)}={}_{\:}{}^{\text{e}\text{x}\text{p}}\mathbf{V}-{{}_{\:}{}^{\text{s}\text{i}\text{m}}\mathbf{V}}^{\left(k\right)}$ is the difference between $\:{}_{\:}{}^{\text{e}\text{x}\text{p}}\mathbf{V}$ and simulated voltage $\:{{}_{\:}{}^{\text{s}\text{i}\text{m}}\mathbf{V}}^{\left(k\right)}$ from the forward simulation with $\:{\varvec{\upsigma\:}}^{\left(k\right)}$, and $\:\lambda\:$ is a hyperparameter which determines the strength of regularization by the L-curve method^22^. In the initial condition setting, this study refers to the absolute conductivity reconstruction algorithm^23^, which ensures that the reconstructed conductivity retains the unit of [S/m]. As shown in Fig. 1**(a)**, $\:\mathbf{J}$ is recalculated and updated at each iteration, which makes this algorithm non-linear with respect of $\:\mathbf{J}$. This approach results in a more accurate inspection of internal morphological structure compared to a linear system. By repeating the algorithm until the convergence of $\:\varDelta\:{\mathbf{V}}^{\left(k\right)}$, $\:\varvec{\upsigma\:}$ was reconstructed.

### Fuzzy phase classification

Figure 1**(b)** shows the fuzzy phase classification which is performed to image air bubble dispersion for understanding the internal morphological structure of whipping cream based on the reconstructed $\:\sigma\:$. In details, each mesh element is clustered into a O/W phase of and air bubble based on $\:\sigma\:$ by the following equations^24^.

6 $$\:{\mu\:}_{j}^{\left(k\right)}=\sum\:_{i=1}^{M}{\left({u}_{ji}\right)}^{h}{\sigma\:}_{i}/\sum\:_{i=1}^{M}{\left({u}_{ji}\right)}^{h}$$

7 $$\:{u}_{ji}={\left\{{\sum\:_{l=1}^{2}\left(\raisebox{1ex}{$\Vert{\sigma\:}_{i}-{\mu\:}_{j}^{\left(k\right)}\Vert$}\!\left/\:\!\raisebox{-1ex}{$\Vert{\sigma\:}_{i}-{\mu\:}_{l}^{\left(k\right)}\Vert$}\right.\right)\:}^{\frac{2}{h-1}}\right\}}^{-1}$$

8 $$\:{\zeta\:}^{\left(k\right)}=\sum\:_{j=1}^{2}\sum\:_{i=1}^{M}{\left({u}_{ji}\right)}^{h}{\Vert{\sigma\:}_{i}-{\mu\:}_{j}^{\left(k\right)}\Vert}^{2}$$

where $\:{\mu\:}_{j}$ is the centroid of cluster, $\:i$ is the mesh index, $\:j=1,\:2$ is the cluster index ($\:j=1$ means O/W phase and $\:j=2$ means air bubble), $\:k$ is the iteration number, $\:h\:(1\le\:h)$ is the hyperparameter which determines fuzziness of softness, $\:\zeta\:$ is the loss function, $\:{\sigma\:}_{i}$ is the conductivity of the *i*-th mesh, and $\:{u}_{ji}$ ($\:0\le\:{u}_{ji}\le\:1$) is the probabilistic cluster of *i*-th mesh. The $\:{u}_{ji}$ is computed by Eqs. (6) and (7). According to $\:{u}_{ji}$, each mesh is probabilistically reclassified into the probabilistic air bubble cluster $\:{u}_{2,i}$. This calculation is repeated iteratively to obtain the probabilistic air bubble cluster $\:{u}_{2}$ until loss function $\:\zeta\:$ is converged. Finally, the spatial mean probabilistic air bubble cluster $\:\langle{u}_{2}\rangle\:$is calculated by the following Equation 

9 $$\:\langle{u}_{2}\rangle={\sum\:}_{i=1}^{M}{\left({u}_{2,i}\right)}^{\:}{A}_{i}/{\sum\:}_{i=1}^{M}{A}_{i}$$

where $\:{A}_{i}$ is the area of $\:i$*-*th mesh.

### Internal morphological structure during agitation

Figure 1**(c)** shows the changes in Internal morphological structure of whipping cream during agitation. Stage 0 represents the state of whipping cream before agitation, Stage 1 represents the period from the start of agitation until the overrun reaches its maximum value and Stage 2 represents the state of the whipping cream after the overrun has reached its maximum value. In Stage 0, the whipping cream consists of water and fat globules, which are dispersed uniformly throughout the water phase due to the adhesion of hydrophilic casein to the fat globules. This uniform dispersion is referred to as the oil-in-water (O/W) phase. In Stage 1, air bubbles introduced by agitation are surrounded by fat globules, which leads to an increase in the overall volume of the whipped cream. As hydrophilic casein is removed during agitation, the exposed hydrophobic fat globules adhere to the air bubble surfaces, resulting in varied air bubble sizes, with larger ones being unstable and prone to collapse. In Stage 2, continued agitation breaks down the larger air bubbles from Stage 1, causing the fat globules that adhered to them to attach to newly incorporated air bubbles, with adhesion occurring more rapidly, resulting in smaller, more uniform, and stable air bubbles, which reduce the overrun. Additionally, the fat globules bond with each other to form a network structure, which stabilizes the air bubbles and enhances the overall stability of the whipped cream^25^. Stages 0, 1, and 2 display the changes in air bubble dispersion during agitation. Based on the differences in conductivity between the O/W phase (10^−1^ S/m) and air bubbles (10^−15^ S/m), *f*EIT is utilized to separate the air bubbles from the O/W phase in the whipping cream. The *f*EIT enables the imaging of air bubble dispersion, where the O/W phase is represented in black and the air bubbles in white.

## Experiments

### fEIT experiment

#### Experimental setup

**Fig. 2:**
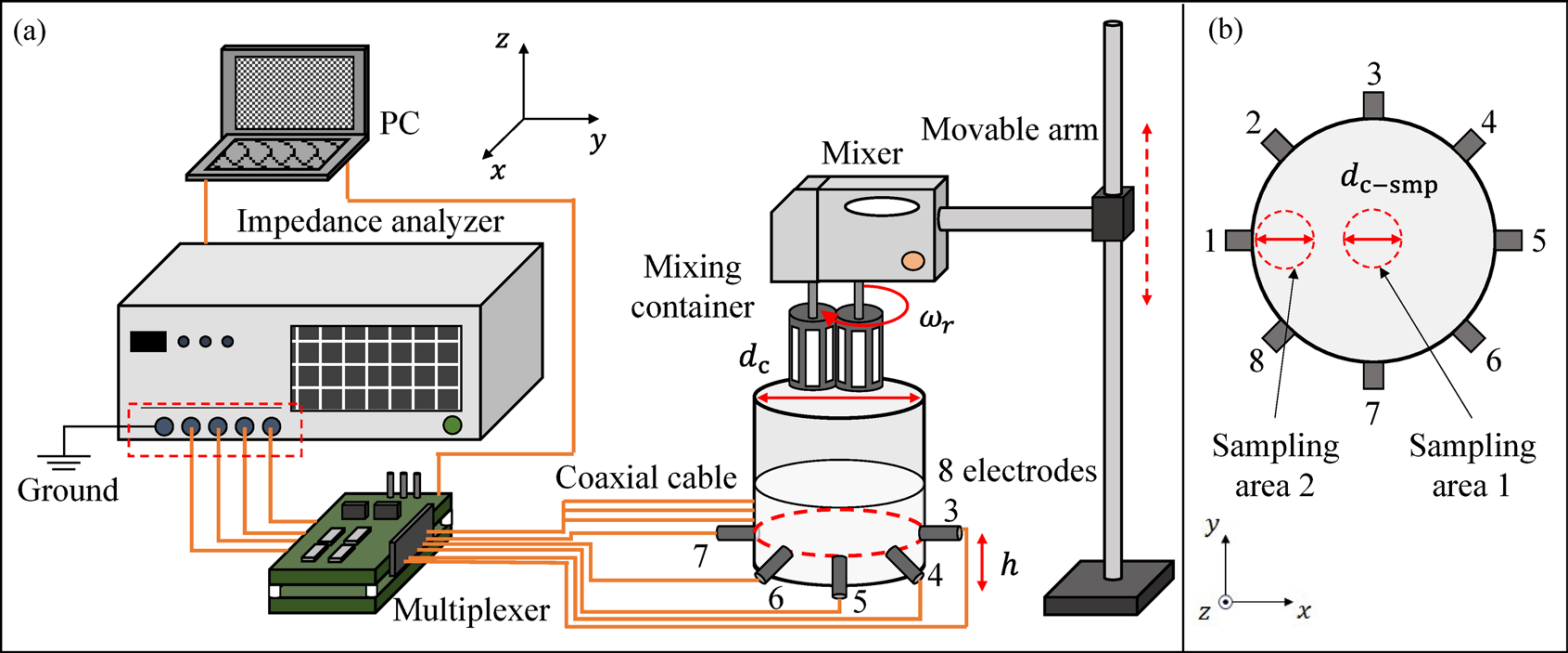
Experimental setup. (**a**) EIT measurement. (**b**) Cross-section of the mixing container.

Figure 2 shows the experimental setup. Figure 2 (a) shows the experimental setup, which includes an impedance analyzer (IM3570, HIOKI E. E., Japan), a multiplexer, a mixing container with electrodes, a mixer (MK-H4, Panasonic, Japan), and a PC. The impedance analyzer, which has two ports for constant current injections (high current HC and low current LC), and two ports for voltage measurements (high potential HP and low potential LP), was employed to measure the impedance. These four ports were connected to the multiplexer’s ports by coaxial cables. The multiplexer was employed to switch electrical signals onto different electrodes with the applied measurement pattern. The mixing container (a cylindrical tank with a diameter of $\:{d}_{c}=84$ mm), which has eight electrodes evenly placed around a cross-section at a height of $\:h=20$ mm from the bottom, was employed for agitation. The mixer with two metal stirring blades was fixed to a vertically movable arm and easily removed from inside the mixing container. PC controlled the impedance analyzer and the multiplexer. Figure 2 (b) shows the cross-section of the mixing container at $\:h=20$ mm. On the cross-section, two circular sampling areas with a diameter *d*_c−smp_ of 20 mm are designated for OR measurements and microscope observations. Sampling area 1 was located at the center of the cross-section and Sampling area 2 was positioned near the wall at electrode 1.

#### Experimental methods

**Fig. 3:**
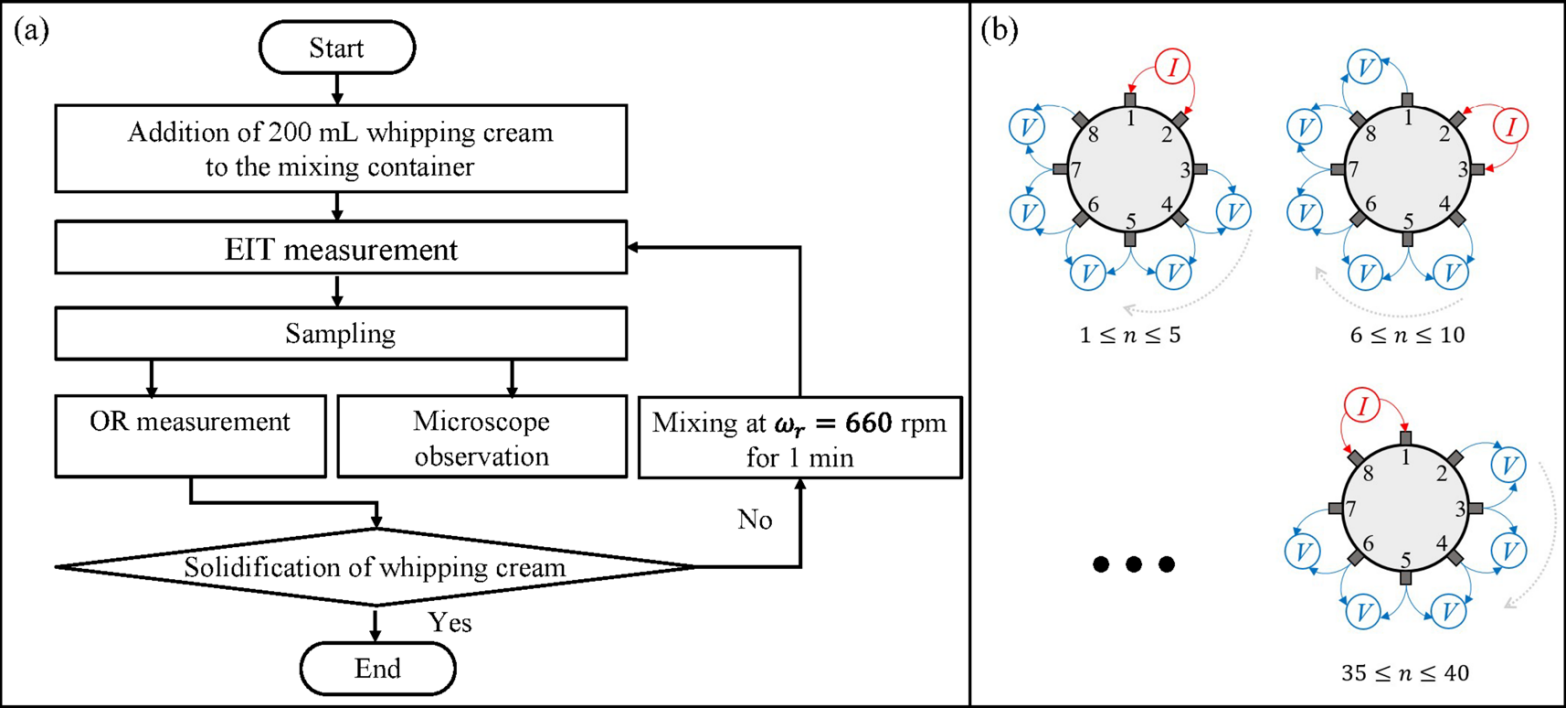
Experimental method. (**a**) Method flowchart. (**b**) Adjacent method with 8 electrodes.

Figure 3 shows the experimental method. Figure 3 (a) shows the experimental method’s flowchart, which includes EIT measurement, sampling, overrun ($\:OR$) measurement, microscope observation and agitation. To verify reproducibility, the entire experiment was repeated three times, with each trial continuing until the whipping cream solidified, as determined by the *OR* results. For the EIT measurements, the adjacent method was applied, which injected a constant alternating current (AC) between one pair of electrodes and measured the potential difference between different pairs of electrodes^26^. Figure 3 (b) shows the details of the adjacent method with 8 electrodes, where the number of the measurement pattern is $\:N=40$. The measured voltage $\:{}_{\:}{}^{\text{e}\text{x}\text{p}}V$was employed to reconstruct the air bubble distribution at different times during agitation by *f*EIT.

#### Experimental conditions

Five types of commercial whipping creams with different milk fat (MF) contents were measured in the experiments, which were MF25 (MF 25%, Nakazawa, Japan), MF35 (MF 35%, Megmilk Snow Brand, Japan), MF40 (MF 40%, Megmilk Snow Brand, Japan), MF43 (MF 43%, Megmilk Snow Brand, Japan) and MF47 (MF 47%, Megmilk Snow Brand, Japan). Before the experiment, the whipping cream was kept in the refrigerator at a temperature of 5 °C. During the experiment, the whipping cream was kept at a temperature range of 4.6 to 6.3°, as fat globules are easily damaged over the temperature of 10 °C^27^. For the EIT measurement, the amplitude of the injection current was set to $\:I=0.1$ mA, the frequency was $\:f=$ 10 kHz and the measurement was repeated three times to obtain the average value. For the agitation, the rotation speed was *ω*_*r*_ = 660 rpm, and the mixing duration was 1 min.

### Overrun (OR) and microscopy experiments

OR measurements were performed to measure the state of whipping cream during agitation by quantitatively evaluating the air incorporation through the ratio of volume change before and after agitation^28^. In this study, $\:OR$ [%] is quantitatively calculated based on the weight of before and after agitation with a fixed volume, which is expressed as follows:

11 $$\:OR=\frac{{m}_{0}-{m}_{{n}_{t}}}{{m}_{{n}_{t}}}\times\:100$$

where $\:{m}_{0}$ [g] is the weight of the whipping cream at a fixed volume prior to agitation, $\:{m}_{{n}_{t}}$ [g] is the weight of the whipping cream at a fixed volume after *n*_*t*_-th agitation and *n*_*t*_ is the index of agitation time. In $\:OR$ measurements, the weight of the whipping cream at a sampling volume of 10 ml was measured by a Semi-Micro Analytical Balance (GH-120, A&D Company, Limited, Japan), which has a minimum readable value of 0.1 mg. This balance provides excellent measurement accuracy and reproducibility, ensuring the reliability of the measurements. Multiple samples are taken from both Sample area 1 (at the center) and 2 (near the wall) as shown in Figure. 2 (b) to ensure consistency.

Microscope observations were performed to analyse the structure change of whipping cream during agitation. An optical microscope (ECLIPSE Ti2-E, Nikon, Japan) equipped with a 10x objective lens (Plan Fluor 10x OFN25 Ph1 DL, Nikon, Japan) was applied to capture the images of the whipping cream samples. The samples were prepared by taking 2 ml of the whipping cream, which was then placed between two cover glasses (C015001, Matsunami Glass Ind, Japan) for observation.

## Results and discussions

### OR results

**Fig. 4:**
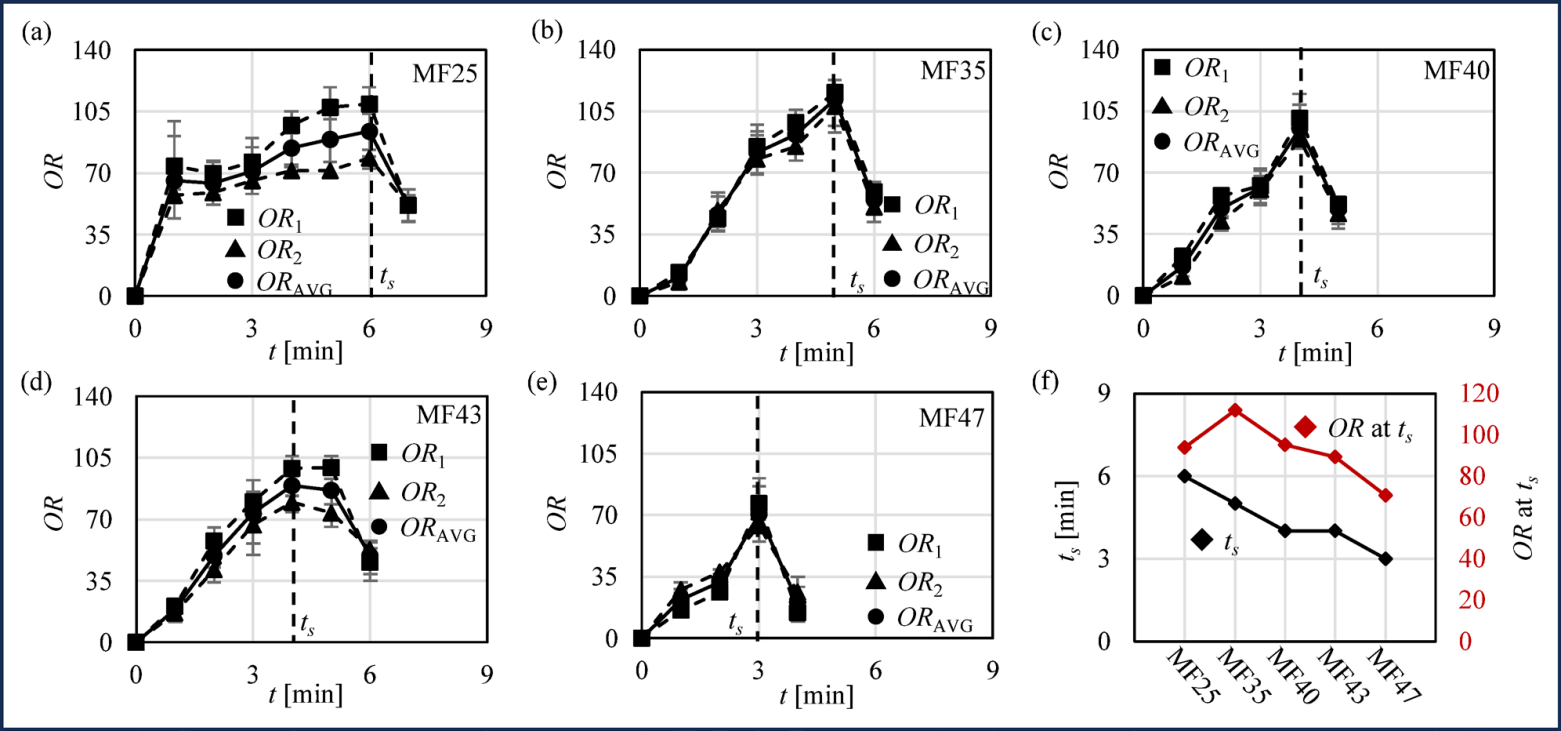
OR results. (**a**)~(**e**) *OR*_1_ (average of three measurements) in Sample area 1, *OR*_2_ (average of three measurements) in Sample area 2 and overall average *OR*_AVG_ with error bar of MF25, MF 35, MF40, MF43 and MF47 over time. (**f**) Peak *OR*_AVG_ and time *t*_*s*_ of peak *OR*_AVG_ of MF25, MF 35, MF40, MF43 and MF47.

$\:OR$ results provide insights into the extent of air bubble incorporation into whipping cream during agitation and indicate the progress of whipping cream toward its critical saturation point, which reflects the stability and dispersion of air bubbles in the whipping cream. Figure 4 shows $\:OR$ over time, where $\:O{R}_{1}$ and $\:O{R}_{2}$ represent the averages of three measurements taken from Sampling area 1 located at the center of the cross-section and Sampling area 2 positioned near the wall at electrode 1 as shown in Fig. 2 (b), and $\:O{R}_{\text{A}\text{V}\text{G}}$ represents the overall average of these six measurements with absolute deviation. Figure 4 (a)~(e) show $\:OR$ of MF25 ~ MF47 over time. In all cases, *OR* first increases due to air bubble incorporation and then decreases as air bubbles escape. Also, $\:O{R}_{1}$ and $\:O{R}_{2}\:$are similar with $\:O{R}_{\text{A}\text{V}\text{G}}$, which suggests uniform agitation. Figure 4**(f)** shows the peak $\:O{R}_{\text{A}\text{V}\text{G}}$ and *t*_*s*_, where the peak $\:O{R}_{\text{A}\text{V}\text{G}}$ represents the critical saturation point of air bubbles and *t*_*s*_ represents the timing of the critical saturation point of air bubbles. The *t*_*s*_ is decreased as the MF content increased (*t*_*s−MF25*_ = 6 s, *t*_*s−MF35*_ =5 s, *t*_*s−MF40*_ = 4 s, *t*_*s−MF43*_ = 4 s and *t*_*s−MF47*_ = 3 s), which is attributed to the enhanced air bubbles stability in whipping cream with higher MF content. As the MF content increases, a stronger layer of partially coalesced fat forms around the air bubbles during agitation, which stabilizes the air bubbles more effectively, accelerates their aggregation, and promotes quicker formation of a solid-like structure^29^. The peak $\:O{R}_{\text{A}\text{V}\text{G}}$ is also decreased as the MF content increased.

**Fig. 5:**
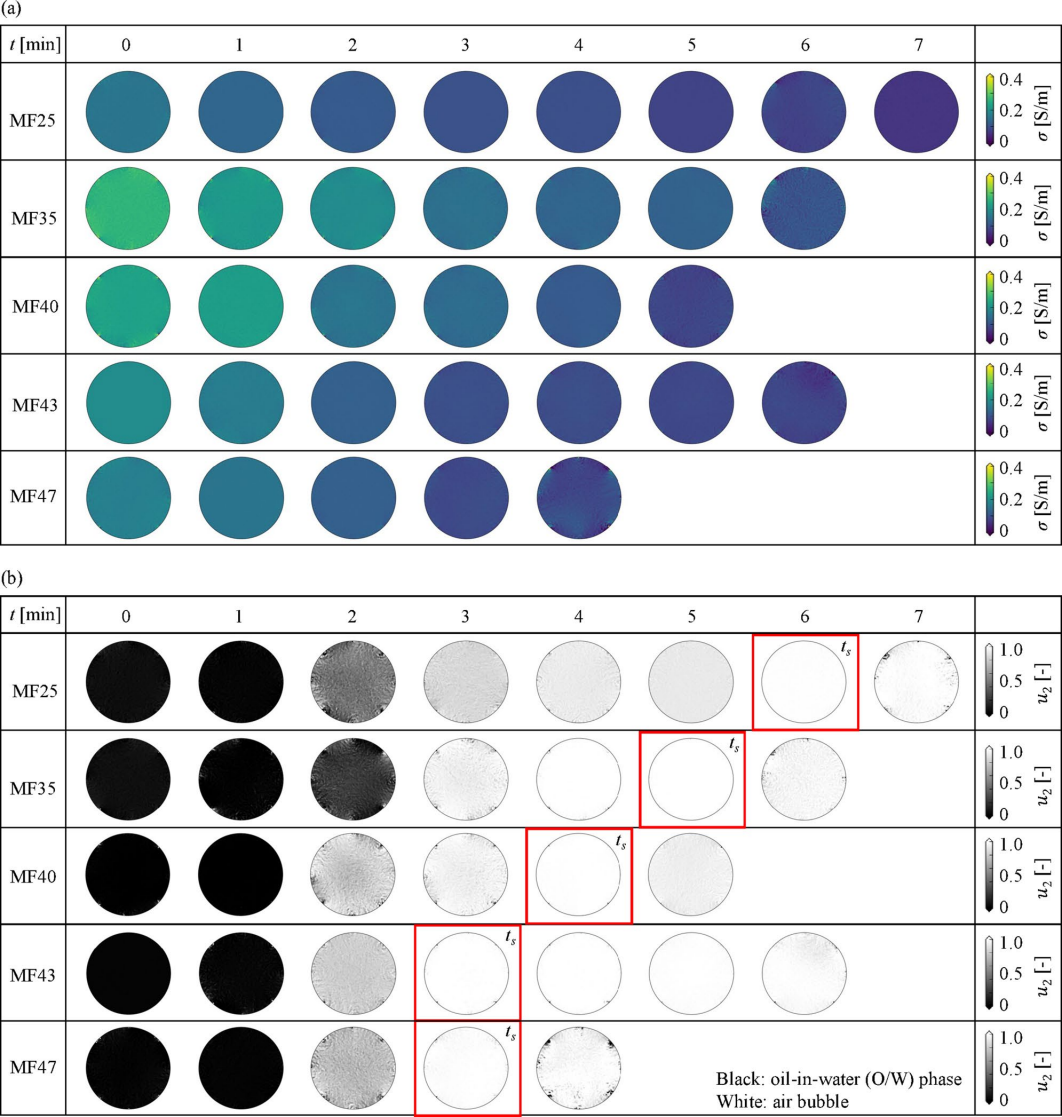
*f*EIT results. (**a**) Reconstructed conductivity distribution *σ* by EIT. (**b**) Calculated probabilistic air bubble cluster $\:{u}_{2}$ by fuzzy phase classification, where black means the oil-in-water (O/W) phase and white means the air bubble.

### fEIT results

Figure 5 shows the *f*EIT results of five types of whipping cream over time during agitation. Figure 5**(a)** shows the conductivity distribution of the whipping cream reconstructed by non-linear conductivity reconstruction as shown in Fig. 1**(a)**, with the electrode orientation matching that shown in Fig. 2 (b). Since the EIT measurements were terminated after solidification, the longest measurement time for MF25 (*t* = 7 min) indicated the latest attainment of the critical saturation point of air bubbles, while the shortest measurement time for MF47 (*t* = 4 min) indicated the shortest attainment of the critical saturation point of air bubbles. As mentioned in OR results, the higher the MF content, the earlier the whipping cream reached the critical saturation point of air bubbles with agitation. For the five types of whipping cream, the conductivity exhibited slight variations, following the same trend before and after reaching the critical saturation point of air bubbles. The reconstructed conductivity distribution based on EIT displays the conductivity variations caused by the distribution of the liquid water phase and air bubbles but does not provide direct information about the exact distribution of these phases. Figure 5 (b) shows the probabilistic air bubble cluster $\:{u}_{2}$ [-] calculated by fuzzy phase classification as shown in Fig. 1 (b). The color bar represented the air bubble percentage, where 0 indicated the absence of air bubbles (O/W phase) and 1 represented the presence of only air bubbles. The $\:{u}_{2}$ was firstly increased and then decreased, which indicated that during agitation, air bubbles continuously entered the whipping cream until reaching the critical saturation point, after which the air bubbles were expelled from the whipping cream. The $\:{u}_{2}$ reconstructed by *f*EIT clearly demonstrated the distribution of the liquid water phase and air bubbles in the whipping cream.

**Fig. 6:**
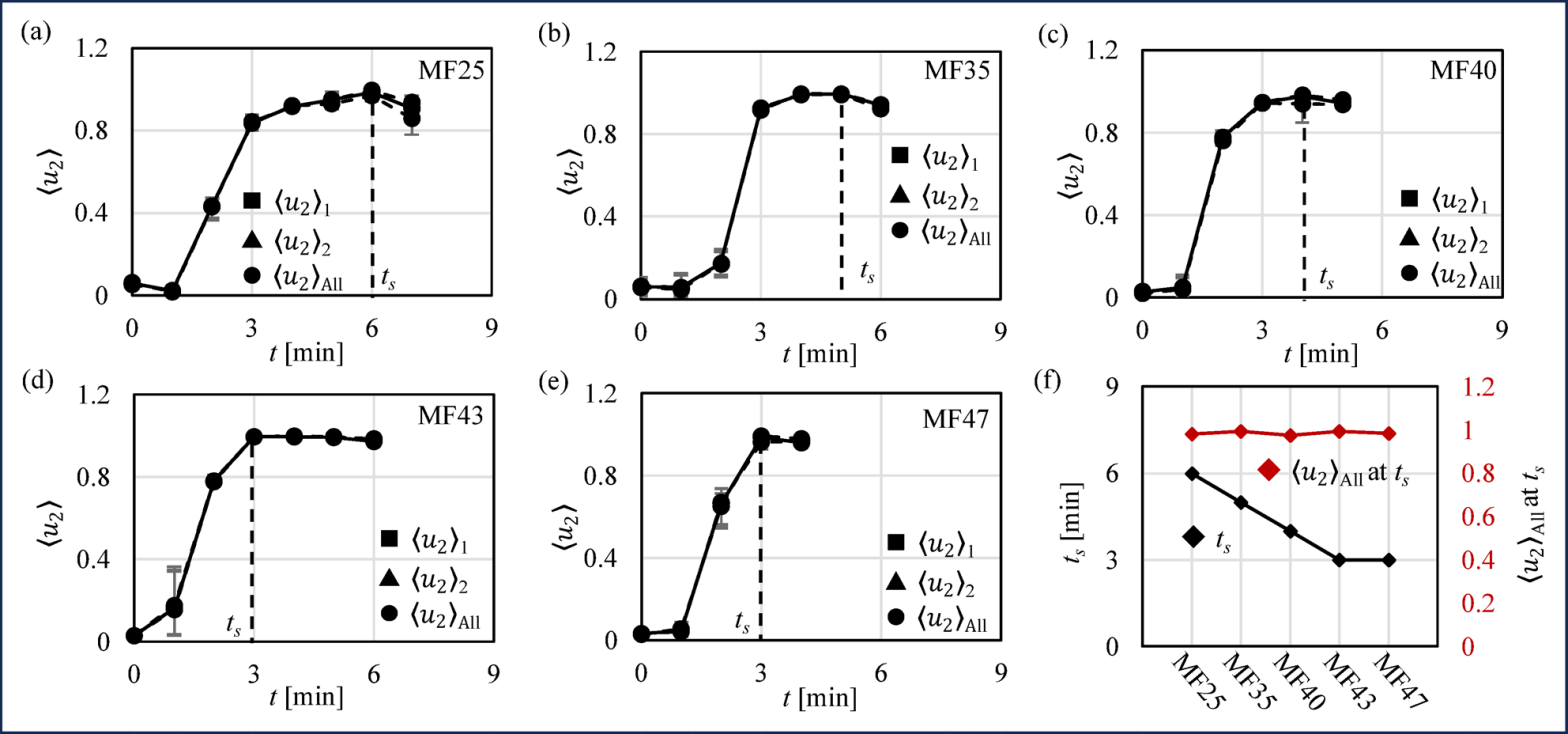
*f*EIT results. (**a**)~(**e**) probabilistic air bubble cluster of sampling area 1 $\:{\langle{u}_{2}\rangle}_{1}$, Sampling area 2 $\:{\langle{u}_{2}\rangle}_{2}$ and all sensor area $\:{\langle{u}_{2}\rangle}_{\text{A}\text{l}\text{l}}$ of MF25, MF35, MF40, MF43 and MF47 over time. (**f**) Peak $\:{\langle{u}_{2}\rangle}_{\text{A}\text{l}\text{l}}$ and time *t*_*s*_ of peak $\:{\langle{u}_{2}\rangle}_{\text{A}\text{l}\text{l}}$ of MF25, MF 35, MF40, MF43 and MF47.

Figure 6 (a)~(e) shows the average probabilistic air bubble cluster of sampling area 1 (at the center) $\:{\langle{u}_{2}\rangle}_{1}$, sampling area 2 (near the wall) $\:{\langle{u}_{2}\rangle}_{2}$ and all sensor area $\:{\langle{u}_{2}\rangle}_{\text{A}\text{l}\text{l}}$ over time, with the error bars representing absolute deviation. Similar to the $\:O{R}_{\:}$results, the similarity of $\:{\langle{u}_{2}\rangle}_{1}$, $\:{\langle{u}_{2}\rangle}_{2}$ and $\:{\langle{u}_{2}\rangle}_{\text{A}\text{l}\text{l}}$ of all cases demonstrates uniform agitation. In all cases, $\:{\langle{u}_{2}\rangle}_{1}$, $\:{\langle{u}_{2}\rangle}_{2}$ and $\:{\langle{u}_{2}\rangle}_{\text{A}\text{l}\text{l}}$ first increases due to air bubble incorporation and then decreases as air bubbles escape. The *t*_*s*_ was decreased as the MF content increased (*t*_*s−MF25*_ = 6 s, *t*_*s−MF35*_ =5 s, *t*_*s−MF40*_ = 4 s, *t*_*s−MF43*_ = 3 s, *t*_*s−MF47*_ = 3 s). Figure 6**(f)** shows the peak $\:{\langle{u}_{2}\rangle}_{\text{A}\text{l}\text{l}}$ and *t*_*s*_ of all cases. The *t*_*s*_ is decreased as the MF content increased (*t*_*s−MF25*_ = 6 s, *t*_*s−MF35*_ =5 s, *t*_*s−MF40*_ = 4 s, *t*_*s−MF43*_ = 3 s and *t*_*s−MF47*_ = 3 s), which shows the same trend as OR results. The peak $\:{\langle{u}_{2}\rangle}_{\text{A}\text{l}\text{l}}$ of five cases were nearly identical, approaching 1. This is because, to preserve the temporal dimension of $\:\langle{u}_{2}\rangle$, conductivity images of all time points for each condition were used as inputs for fuzzy phase classification. Through the fuzzy phase classification, $\:\langle{u}_{2}\rangle$ approached 0 when air bubble content was low and approached 1 when air bubble content was high. Consequently, at the critical saturation point of air bubbles, $\:{\langle{u}_{2}\rangle}_{\text{A}\text{l}\text{l}}$ of all cases are similar and close to 1.

### Microscopy results

**Fig. 7:**
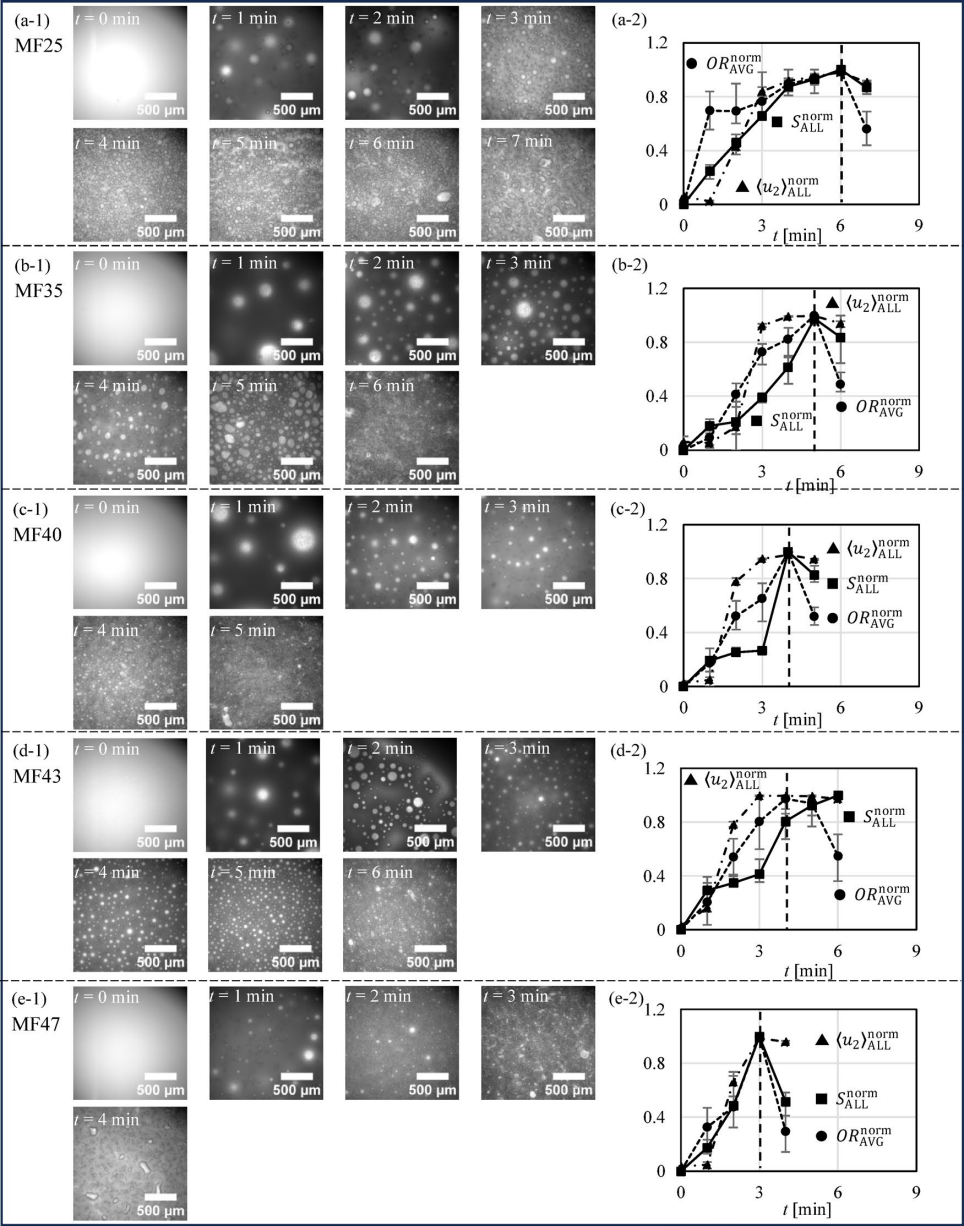
Microscopy results. (**a-1**)~(**e-1**) Microscopy images of MF25, MF35, MF40, MF43 and MF47 over time. (a-2)~(e-2) Normalized average $\:{OR}_{\text{A}\text{V}\text{G}}^{\text{n}\text{o}\text{r}\text{m}}$, normalized average probabilistic air bubble cluster $\:{\langle{u}_{2}\rangle}_{\text{A}\text{L}\text{L}}^{\text{n}\text{o}\text{r}\text{m}}$ and normalized air bubble area ratio $\:{S}_{\text{R}\text{A}\text{T}\text{I}\text{O}}^{\text{n}\text{o}\text{r}\text{m}}$ of MF25, MF35, MF40, MF43 and MF47 over time.

Figure 7 shows the microscopy results of MF25, MF35, MF40, MF43 and MF47. Figure 7 (a-1)~(e-1) shows the microscopy images of whipping cream over time. For MF25 as an example, at *t* = 0 min (before agitation), there were no air bubbles in the whipping cream. During agitation from *t* = 1 min to 6 min, air bubbles gradually incorporated into the whipping cream, with a decrease in air bubble size and an increase in are bubble number. At *t* = 7 min (overmixed), phase separation into liquid and solid phases occurred due to the excessive clumping of fat globules, which broke the emulsion^30^. Figure 7 (a-2)~(e-2) shows the normalized average $\:{OR}_{\text{A}\text{V}\text{G}}^{\text{n}\text{o}\text{r}\text{m}}$, normalized average probabilistic air bubble cluster $\:{\langle{u}_{2}\rangle}_{\text{A}\text{L}\text{L}}^{\text{n}\text{o}\text{r}\text{m}}$ and normalized air bubble area ratio $\:{S}_{\text{R}\text{A}\text{T}\text{I}\text{O}}^{\text{n}\text{o}\text{r}\text{m}}$ of MF25, MF35, MF40, MF43 and MF47, with the error bars representing absolute deviation. The $\:{OR}_{\text{A}\text{V}\text{G}}^{\text{n}\text{o}\text{r}\text{m}}$ and $\:{\langle{u}_{2}\rangle}_{\text{A}\text{L}\text{L}}^{\text{n}\text{o}\text{r}\text{m}}$ were calculated by normalizing $\:{OR}_{\text{A}\text{V}\text{G}}^{\:}$ shown in Fig. 4 and $\:{\langle{u}_{2}\rangle}_{\text{A}\text{L}\text{L}}^{\:}$ shown in Fig. 6 with the following equations to normalized the data to a range between 0 and 1.

12 $$\:{OR}_{\text{A}\text{V}\text{G}}^{\text{n}\text{o}\text{r}\text{m}}\left({n}_{t}\right)=\frac{{OR}_{\text{A}\text{V}\text{G}}^{\:}\left({n}_{t}\right)-\text{min}\left({OR}_{\text{A}\text{V}\text{G}}^{\:}\right)}{\text{max}\left({OR}_{\text{A}\text{V}\text{G}}^{\:}\right)-\text{m}\text{i}\text{n}\left({OR}_{\text{A}\text{V}\text{G}}^{\:}\right)}$$

13 $$\:{\langle{u}_{2}\rangle}_{\text{A}\text{L}\text{L}}^{\text{n}\text{o}\text{r}\text{m}}\left({n}_{t}\right)=\frac{{\langle{u}_{2}\rangle}_{\text{A}\text{L}\text{L}}^{\:}\left({n}_{t}\right)-\text{min}\left({\langle{u}_{2}\rangle}_{\text{A}\text{L}\text{L}}^{\:}\right)}{\text{max}\left({\langle{u}_{2}\rangle}_{\text{A}\text{L}\text{L}}^{\:}\right)-\text{m}\text{i}\text{n}\left({\langle{u}_{2}\rangle}_{\text{A}\text{L}\text{L}}^{\:}\right)}$$

where $\:{n}_{t}$ [-] is the index of time, $\:\text{min}\left({OR}_{\text{A}\text{V}\text{G}}^{\:}\right)$ and $\:\text{max}\left({OR}_{\text{A}\text{V}\text{G}}^{\:}\right)$ are the minimum and maximum values of $\:{OR}_{\text{A}\text{V}\text{G}}^{\:}$, respectively, and similarly for $\:{\langle{u}_{2}\rangle}_{\text{A}\text{L}\text{L}}^{\:}$.

The $\:{S}_{\text{R}\text{A}\text{T}\text{I}\text{O}}^{\text{n}\text{o}\text{r}\text{m}}$ was calculated by $\:{S}_{\text{a}\text{i}\text{r}}/{S}_{\text{A}\text{L}\text{L}}$, where $\:{S}_{\text{a}\text{i}\text{r}}$ is the total area of air bubbles in the microscopy images and $\:{S}_{\text{A}\text{L}\text{L}}$ is the total area of the microscopy images, which is 3.159862 × 10^6^ µm^2^ (1777.6 μm × 1777.6 μm). The $\:{S}_{\text{a}\text{i}\text{r}}$ was calculated with ImageJ software^31^ by analyzing the microscopy images. In Fig. 7 (a-2)~(e-2), $\:{OR}_{\text{A}\text{V}\text{G}}^{\text{n}\text{o}\text{r}\text{m}}$ and $\:{S}_{\text{R}\text{A}\text{T}\text{I}\text{O}}^{\text{n}\text{o}\text{r}\text{m}}$ are considered the most accurate indicator of air bubble quantity in whipping cream, with observable immediately upon initial agitation (at *t* = 1 min) due to entry of air bubbles. The $\:{\langle{u}_{2}\rangle}_{\text{A}\text{L}\text{L}}^{\text{n}\text{o}\text{r}\text{m}}$ at 1 min were similar to those at 0 min, which indicates that $\:{\langle{u}_{2}\rangle}_{\text{A}\text{L}\text{L}}^{\text{n}\text{o}\text{r}\text{m}}$ is insensitive to low air bubble content. From *t* = 2 min, $\:{S}_{\text{R}\text{A}\text{T}\text{I}\text{O}}^{\text{n}\text{o}\text{r}\text{m}}$, $\:{OR}_{\text{A}\text{V}\text{G}}^{\text{n}\text{o}\text{r}\text{m}}$, $\:{\langle{u}_{2}\rangle}_{\text{A}\text{L}\text{L}}^{\text{n}\text{o}\text{r}\text{m}}$ are increased due to an increase in air bubble percentage, which is also consistent with the microscopy images. For MF25 as an example, at *t* = 6 min, a critical saturation point of air bubbles was reached for all $\:{\langle{u}_{2}\rangle}_{\text{A}\text{L}\text{L}}^{\text{n}\text{o}\text{r}\text{m}}$, $\:{OR}_{\text{A}\text{V}\text{G}}^{\text{n}\text{o}\text{r}\text{m}}$ and $\:{S}_{\text{R}\text{A}\text{T}\text{I}\text{O}}^{\text{n}\text{o}\text{r}\text{m}}$, which was evidenced by the subsequent decrease after *t* = 6 min.

### Conductivity variations of whipping cream during agitation

**Fig. 8:**
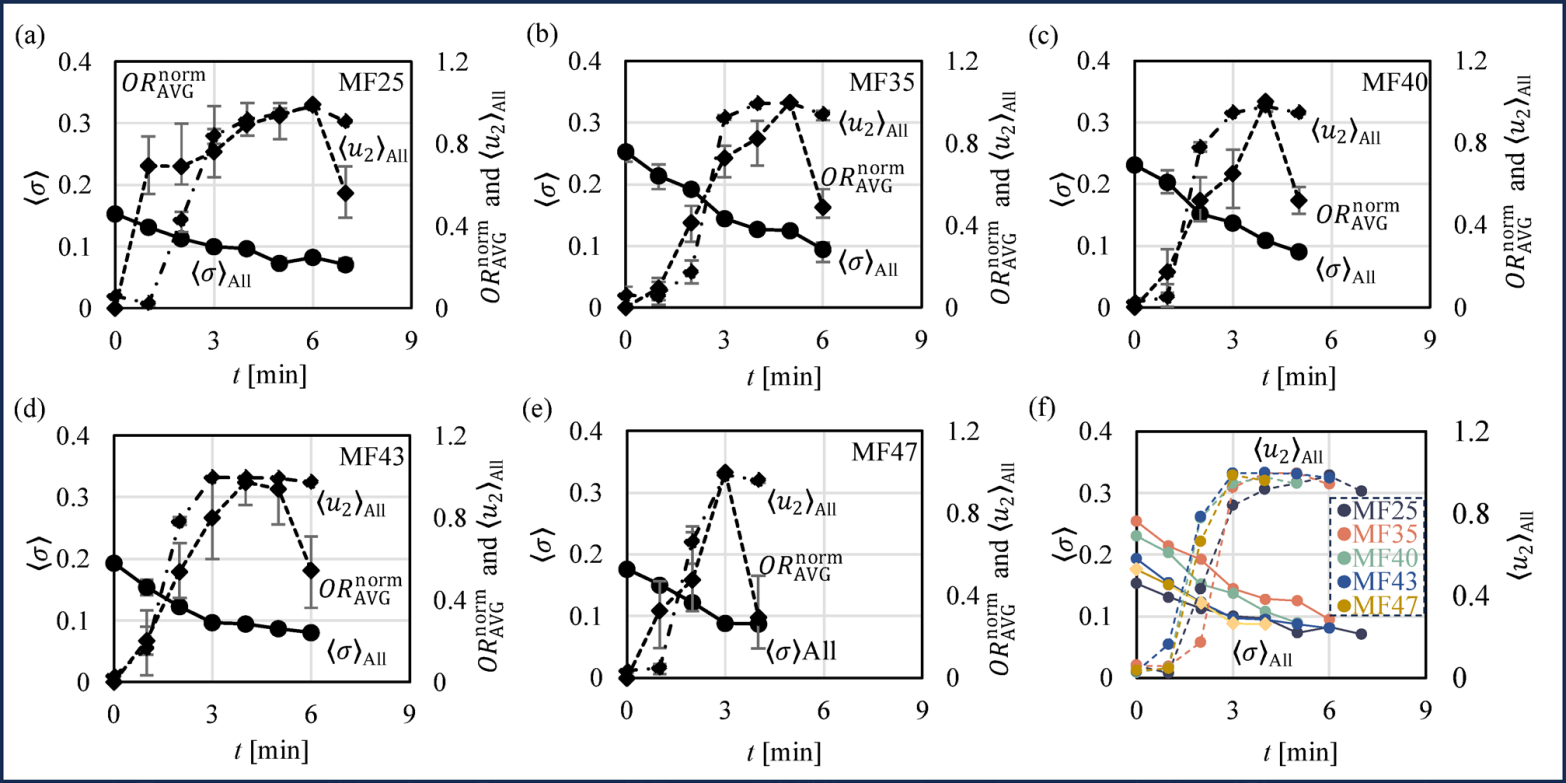
Spatial average conductivity 〈σ〉 by EIT. (**a**)~(**e**) Reconstructed 〈σ〉_All_ of all sensor area, normalized average $\:{OR}_{\text{A}\text{V}\text{G}}^{\text{n}\text{o}\text{r}\text{m}}$, and probabilistic air bubble cluster $\:{\langle{u}_{2}\rangle}_{\text{A}\text{L}\text{L}}^{\:}$ of all sensor area of MF25, MF 35, MF40, MF 43 and MF47 over time. (**f**) Comparison of 〈σ〉_All_ and $\:{\langle{u}_{2}\rangle}_{\text{A}\text{l}\text{l}}$ over time.

Figure 8 shows the conductivity of whipping cream reconstructed by EIT. Figure 8**(a)~(e)** show the spatial average conductivity of all sensor area 〈σ〉_All_, Sample area 1 〈σ〉_1_ and Sample area 2 〈σ〉_2_ of MF25, MF35, MF40, MF43 and MF47 over time, where Sample area 1 (at the center) and 2 (near the wall) are shown in Fig. 2 (b). Since 〈σ〉_1_, 〈σ〉_2_ and 〈σ〉_All_ are essentially consistent, only 〈σ〉_All_ is indicated in figures. The results show that 〈σ〉_All_ was decreased during agitation under all MF conditions. The observed gradual decrease in 〈σ〉_All_ during agitation of whipping cream is attributed to the increasing presence of air bubbles within the emulsion^32^, which is closely linked to changes in the internal morphological structure and material composition of the whipping cream during agitation. Firstly, as air bubbles are introduced into the whipping cream, the formation of air bubbles leads to a progressive replacement of the conductive liquid phase by the non-conductive air phase. As air bubbles, which are electrical insulators with conductivities near zero, increase in volume fraction, air bubbles significantly reduce the overall conductivity of the system^33^. Secondly, the dynamic behavior of fat globules during agitation plays a critical role. As the agitation progresses, fat globules aggregate and partially coalesce around air bubbles, which form a thin and insulate fat layer at the air-bubble interface. This partially coalesced fat network further inhibits conductivity by reducing the effective electrical pathways through the continuous liquid phase^34^. Additionally, the structural transformation of the whipping cream from a liquid-like emulsion to a more solid-like structure also contributes to the decrease in conductivity. During the transition, the distribution of the liquid phase becomes increasingly heterogeneous, and the conductive liquid bridges between air bubbles become less prominent, which disrupts the pathways for electrical current^35^. Future studies could further explore the relationship between the conductivity decrease and the fat content of the whipping cream, as well as investigate the potential for optimizing agitation parameters to achieve specific air bubble incorporation targets. Such insights would not only enhance the understanding of whipping cream dynamics but also improve quality control in food processing applications.

### Whipped cream internal morphological structure evaluation by fEIT compared to conventional EIT

As illustrated qualitatively in Fig. 5 (a) by σ, and further quantified in Fig. 8 (a)–(f) through 〈σ〉, evaluating the internal morphological structure of whipped cream proves to be challenging. In this context, the critical saturation point cannot be identified using either the conductivity distribution σ or the spatial average conductivity 〈σ〉 in conventional electrical impedance tomography (EIT). Specifically, the air bubbles, which represent the air phase, are not clearly discernible in σ distribution. The σ suggests a diffuse mixture of the O/W phase and air bubble, making the air bubble visualization unclear. Furthermore, as confirmed by the trend of 〈σ〉 across various milk fat (MF) cases, the trendline primarily reflects the average air bubble content over time with a negative gradient. Therefore, it is not possible to precisely determine the critical saturation point at any given time *t* [min] using conventional EIT. In contrast, as shown qualitatively in Fig. 5**(b)** by σ, and quantitatively validated in Fig. 8 (a)–(f) through $\:\langle{u}_{2}\rangle$, *f*EIT provides a more effective approach for assessing the internal morphological structure of whipped cream and identifying the critical saturation point at a specific time *t*. In this case, the air bubbles are clearly visible as the white areas in the reconstructed image of the probabilistic air bubble cluster $\:{u}_{2}$, obtained through fuzzy phase classification. The trend of $\:\langle{u}_{2}\rangle$, verified by the $\:OR$ for each MF case, shows a positive gradient over time, which subsequently transitions to a negative gradient at a specific time *t*. As a result, *f*EIT enables a clear identification of the critical saturation point at a given time *t* for each MF case. Thus, *f*EIT demonstrates its superiority over conventional EIT in accurately determining the critical saturation point of whipped cream during agitation.

## Conclusions

In this study, air bubble dispersion in whipping cream with various fat contents was imaged by fuzzy phase classification implemented in electrical impedance tomography (*f*EIT).

1. The *f*EIT, proposed by implementing fuzzy phase classification in electrical impedance tomography, successfully imaged the air bubble dispersion in the whipping cream by reconstructing the probabilistic air bubble cluster $\:{u}_{2}$.
2. The $\:{u}_{2}$ of whipping cream with five milk fat (MF) contents were reconstructed by *f*EIT during agitation to validate the air bubbles dispersion. The results showed that the peak time *t*_*s*_ of the spatial average $\:{\langle{u}_{2}\rangle}_{\text{A}\text{l}\text{l}}$ of all sensor area, which represents the critical saturation point of air bubbles, was decreased as the MF content increased (*t*_*s−MF25*_ = 6 s, *t*_*s−MF35*_ =5 s, *t*_*s−MF40*_ = 4 s, *t*_*s−MF43*_ = 3 s, *t*_*s−MF47*_ = 3 s), which was also verified by $\:OR$ results and microscopy images.
3. Compared to the conductivity reconstructed by EIT, which is influenced by the liquid water phase, fat and air bubbles, $\:\langle{u}_{2}\rangle$ reconstructed by *f*EIT successfully reflected the air bubble dispersion in the whipping cream during agitation. Here, the *f*EIT demonstrated comparable performance to $\:OR$ measurements in evaluating the internal morphological structure of whipping cream during agitation, while providing the advantage of inline measurement. These findings highlight the potential of *f*EIT as a reliable and inline tool for assessing the internal dynamics of whipping cream during agitation.

## Data Availability

All data needed to evaluate the conclusions in the paper are present in the paper. Additional data related to this paper can be requested from Dr. Songshi Li (lisongshi@chiba-u.jp).
